# Rate of force development relationships to muscle architecture and contractile behavior in the human vastus lateralis

**DOI:** 10.1038/s41598-022-26379-5

**Published:** 2022-12-17

**Authors:** Amelie Werkhausen, Øyvind Gløersen, Antoine Nordez, Gøran Paulsen, Jens Bojsen-Møller, Olivier R. Seynnes

**Affiliations:** 1 Department of Physical Performance, Norwegian School of Sport Sciences, Oslo, Norway; 2 SINTEF Digital, Smart Sensors and Microsystems, Oslo, Norway; 3 Movement-Interactions-Performance, MIP, UR 4334, Nantes Université, 44000 Nantes, France; 4 Institut Universitaire de France (IUF), Paris, France; 5 Department of Sports Science and Clinical Biomechanics, University of Southern Denmark, Odense, Denmark

**Keywords:** Physiology, Muscle

## Abstract

In this study, we tested the hypotheses that (i) rate of force development (RFD) is correlated to muscle architecture and dynamics and that (ii) force–length–velocity properties limit knee extensor RFD. Twenty-one healthy participants were tested using ultrasonography and dynamometry. Vastus lateralis optimal fascicle length, fascicle velocity, change in pennation angle, change in muscle length, architectural gear ratio, and force were measured during rapid fixed-end contractions at 60° knee angle to determine RFD. Isokinetic and isometric tests were used to estimate individual force–length–velocity properties, to evaluate force production relative to maximal potential. Correlation analyses were performed between force and muscle parameters for the first three 50 ms intervals. RFD was not related to optimal fascicle length for any measured time interval, but RFD was positively correlated to fascicle shortening velocity during all intervals (r = 0.49–0.69). Except for the first interval, RFD was also related to trigonometry-based changes in muscle length and pennation angle (r = 0.45–0.63) but not to architectural gear ratio. Participants reached their individual vastus lateralis force–length–velocity potential (i.e. their theoretical maximal force at a given length and shortening velocity) after 62 ± 24 ms. Our results confirm the theoretical importance of fascicle shortening velocity and force–length–velocity properties for rapid force production and suggest a role of fascicle rotation.

## Introduction

The ability of skeletal muscle to rapidly increase tension from a relaxed state is integral to tasks involving rapid movements and fast adjustments in balance^1,2^. The measurement of rate of force development (RFD) is increasingly used as an indicator of neuromuscular function for both athletes (e.g.^3^), patients and in aging (e.g.^4,5^). However, the determinants and the physiological factors that influence RFD remain debated in the literature.

Early RFD (within the first 50 ms) of an explosive contraction is primarily determined by the ability to activate motor units maximally and rapidly^6,7^. Although the neural system has a major influence on the rate of force development (RFD), several in vivo studies^8^ and a recently developed computational model^9^ suggest the gradual contribution of intrinsic muscular factors during contraction. Hence, RFD seems to be influenced by parameters such as fibre-type composition^10^, muscle size^11^ and muscle–tendon unit stiffness^12,13^. The influence of architectural arrangement of fibres, another intrinsic muscle property, is however still unclear^8^.

Theoretically, architecture can influence RFD in several ways, related to the muscle’s requirement to increase force development while contracting rapidly and to its interaction with the tendon. Even during fixed-end contractions, the potential slack and viscoelastic properties of tendinous tissue sets variable length- and velocity constraints on muscular contractions, particularly in the toe region of the force–elongation relationship. Muscle architecture and architectural changes may therefore affect force production during this mode of contraction. Firstly, the association between numbers of sarcomeres in-series and fibre contraction velocity^14^ suggests that longer fascicles may favour RFD, by reducing the time to take up slack in elastic elements and by enabling larger force production at any given muscle contraction velocity. Secondly, even if fascicles shorten along several axes, the rate force development may also be influenced by an increase in pennation angle, causing a reduction in force effectively transmitted to the tendon^14,15^. Finally, fascicles rotate about their insertions during certain contractions, allowing them to shorten at a slower pace than the whole muscle^16^ and mimicking the effect that additional in series sarcomeres would have in longer fibres. The ratio of fascicle- to muscle velocity, termed architectural gear ratio (AGR), may be particularly important during rapid contractions, when force production ability is limited^16^. Indeed, evidence suggests that rapid fascicle contraction and submaximal force development are supported by neural factors^17,18^. However, how individuals achieve higher RFD via faster fascicle contractile velocities and changes in AGR is not well understood.

In the quadriceps, Maden-Wilkinson et al.^19^ found no correlation between mean resting fascicle length (averaged over the four heads) and early or late rate of torque development^19^. In contrast, Hager et al.^20^, elegantly showed that gastrocnemius medialis force reaches maximal potential according to the force–velocity relation after 100 ms during an RFD test. However, whether the results obtained in this study from the gastrocnemius can directly be transferred to other muscles is unknown. Despite correlation analyses not being included, the results also suggest that links between RFD and architecture may be found when these variables are estimated from a single muscle. Furthermore, the role of contractile properties for RFD suggests that muscle AGR may influence rapid force production. A recent study did find a relationship between architectural gear ratio and explosive torque ability^13^, despite a relatively low reliability of gear ratio^21,22^. Altogether, muscle architecture and contractile properties seem to influence late RFD, but varying methods used in previous reports, or the lack of correlation analyses currently limit our appraisal of these relations.

The aim of this study was to assess the relationships between RFD and muscle architectural and contractile properties in the vastus lateralis muscle. Our first hypothesis was that RFD was positively related to optimal fascicle length, fascicle shortening velocity, pennation angle change and muscle length change enabling faster force transmission. Additionally, we aimed to determine whether RFD is limited by the vastus lateralis muscle reaching its maximal force production capacity with respect to the fascicle force–length–velocity relationship. Based on recent findings for the gastrocnemius medialis muscle^20^, we hypothesised that muscle force would reach its maximal capacity at a given fascicle length and shortening velocity (i.e., the force–length–velocity potential) from approximately 100 ms after onset of contraction.

## Method

### Participants and experimental protocol

Data were collected from 21 healthy individuals (13 males and 8 females, age: 27 ± 4 years; height: 176 ± 10 cm, body mass: 72 ± 11 kg). All participants had no recent history of lower-limb injury and provided written informed consent to participate in the study. The institutional ethics board at the Norwegian School of Sport Sciences, Norway, approved all protocols and the study was performed in accordance with the Declaration of Helsinki.

All participants completed a familiarisation session of isometric and isokinetic knee extensions (IsoMed2000, D&R Ferstl, Hemau, Germany), with their leg randomly selected for the study. The padded interface between the dynamometer arm and the shin was replaced by a stiffer—custom-made interface to stiffen the system. The custom-made interface consisted of a football shin protector coupled with a 3d printed connector to the dynamometer arm. The data collection day started with a standardised warm up of 10-min cycling and ten submaximal single-leg contractions with increasing intensity. Knee extension torque and vastus lateralis ultrasound scans were recorded subsequently, during a protocol of explosive, isometric contractions at 60° knee angle. Finally, isometric and isokinetic contractions efforts were conducted to record maximal torque.

### Knee extension strength testing

Participants were seated and fastened in a dynamometer chair at a hip angle of 80º using leg and hip straps and shoulder pads. The knee rotation axis was aligned to the dynamometer motor axis, and a custom-made connector with a stiff shin pad interfaced the dynamometer arm with the leg. After the warmup, participants performed five explosive contractions, with instructions to push as fast and as hard as they could, with a strong emphasis on the explosive nature of the contraction. We decided to include explosive contraction tests independently of the tests for maximum torque to avoid the possible interference of instructions on either tests^8,23^. Explosive contractions were performed without preactivation to avoid limitations to reach maximum shortening velocities^24^ and motor unit discharge rates^25^. Trials where artefactual countermovement or pre-activation were observed from torque live recordings were repeated. Maximal isometric torque was measured over six knee angles, 100, 90, 80, 70, 60 and 50° (0° being full extension). Maximum isokinetic torque was obtained at angular velocities of 50, 100, 200, 300 and 400° s^−1^. The order of joint angle positions and velocities was randomised, and participants rested for three minutes between each maximum effort to minimise fatigue.

For all contractions, torque was recorded at a sampling frequency of 1500 Hz, and corrected for gravity and passive torque e.g.^26^. Using the knee joint angle we calculated the angle dependent knee moment arm based on literature based functions, as suggested in^27^. Subsequently, we calculated knee extension force by dividing the joint moment by the moment arm for each data point. Reflective markers were used to estimate sagittal plane knee joint angle on the lateral malleolus, tibial head, lateral epicondyle and trochanter major. Markers were recorded by four cameras at 100 Hz (Qualisys, Gothenburg, Sweden). For the explosive contractions, the onset of torque production was determined by finding the first data point below 1 Nm when going backwards from peak torque (i.e. in chronological order, the latest value under the 1 Nm threshold before peak torque, as discussed in Maffiuletti et al.^8^). Trials with a countermovement torque exceeding 3 Nm before the detected onset were discarded from the analysis. From the remaining trials, the best effort, where force was greatest after 150 ms, was used for further analysis.

### Muscle behaviour measurements

B-mode ultrasound images of the vastus lateralis muscle were recorded using two ultrasound probes (LV7.5/60/96Z LS128 (proximal) and LV8-5N60-A2 ArtUs (distal), Telemed, Vilnius, Lithuania) during all contractions. The probes were held in series by a custom 3D-printed cast, angled by 5° between probes to account for the shape of the thigh (as determined empirically in pilot tests). The probe holder was placed along the thigh with the proximal end of the distal probe at 50% of the muscle length, aligned with muscle fascicles. Ultrasound images were sampled at 116 Hz (distal probe) and 82 Hz (proximal probe), which was the maximal sampling frequency of either machine. A square wave analogue pulse from each ultrasound system was used to synchronise all data.

**Figure 1:**
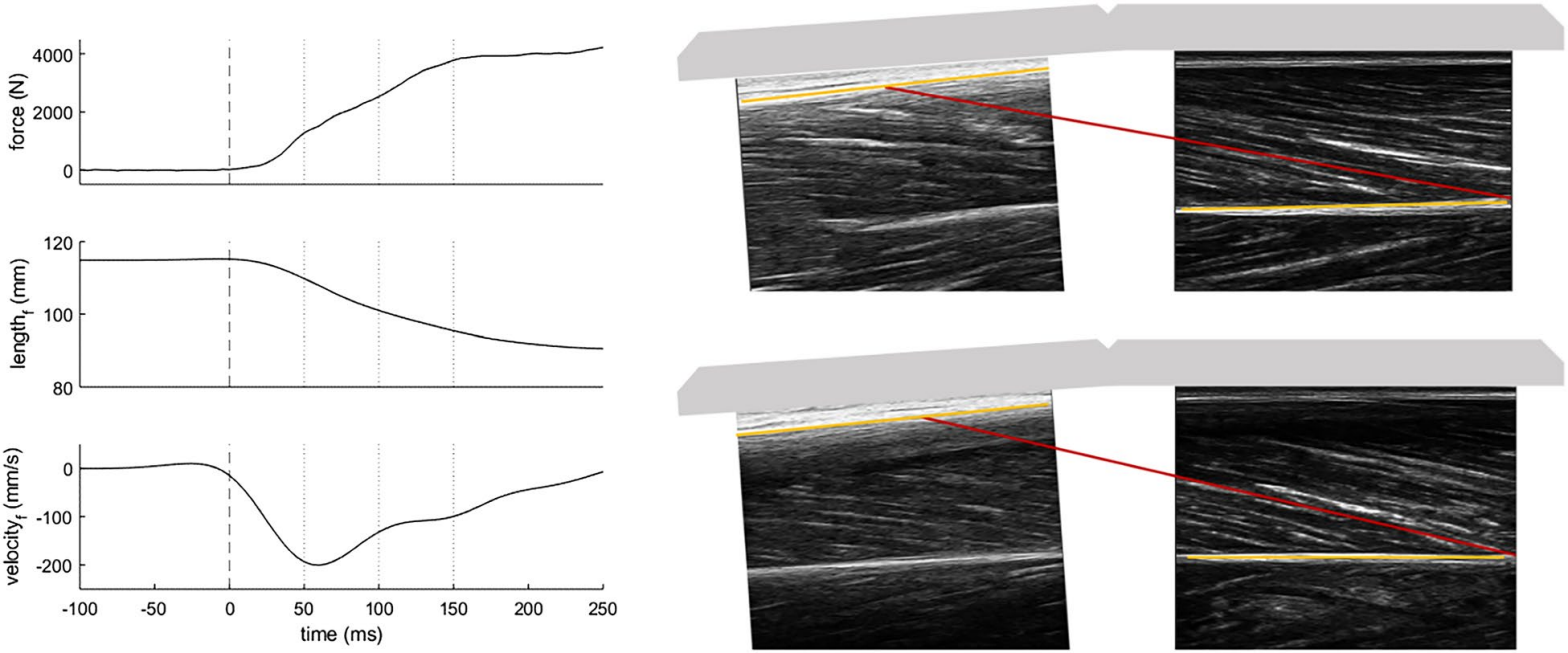
Individual patterns of force, fascicle length (length_f_) and fascicle velocity (velocity_f_) during explosive contractions (left side). Onset of the contraction is shown with the dashed line and 50 ms timepoints/intervals for the analysis are displayed with dotted lines. Changes in force and fascicle length and mean fascicle velocity were calculated for each interval. Example images of longitudinal scans of the vastus lateralis muscle with fascicle length measurements (right side) at force onset (upper panel) and 150 ms after onset (lower panel) with a scaled illustration of the ultrasound transducer holder.

Ultrasound images were digitised using UltraTrack, a semi-automated tracking software^28,29^ to quantify vastus lateralis fascicle length, pennation angle and thickness. Specifically, the position and orientation of the upper- and lower aponeuroses were tracked from the proximal and distal scans, respectively. Fascicle orientation was tracked in the distal scans, as these were collected in the belly of the muscle with the machine offering the highest spatial resolution. Fascicle length was then determined by calculating the distance between the two aponeuroses in the direction of the fascicle orientation (Fig. 1). Pennation angle was calculated as the angle between fascicle orientation and lower aponeurosis orientation. Muscle thickness was defined as the mean distance between the aponeuroses. Muscle length changes were estimated from changes in length of the fascicular projection on the lower aponeurosis, by multiplying fascicle length with the cosine of the pennation angle^30^.

### Modelling of muscle properties

Muscle force–length–velocity properties were estimated by combining models to assess muscle force–length and force–velocity properties^31,32^. We used force, fascicle length and fascicle velocity data from the isometric and isokinetic contractions to produce the individual force–length–velocity profiles based on the force–length (Eq. 1) and force–velocity (Eq. 2) relationships. The experimental data were fitted to the equation using nonlinear least square optimisation:

1 $${F(L)={{F}_{\mathrm{max}}\cdot e}^{-\left|\left(\frac{{\left(\frac{L}{{L}_{0}}\right)}^{b}-1}{s}\right)\right|}}^{2}$$

2 $$F\left(v\right)= \frac{\left(1-\frac{v}{{v}_{max}}\right)}{1+G\cdot v/{v}_{max}}$$

3 $$F= F\left(L\right)\cdot F\left(v \right)$$

where *F* is the force capacity, *F*_max_ is isometric force at optimal fascicle length (*L*_0_), *L* is fascicle length, *b* is the skewness, *s* is the width of the curve, *v* is fascicle shortening velocity, *v*_*max*_ is the maximum shortening velocity and *G* is the curvature parameter. We did not constrain v_max_ in our model because we measured fascicle velocity at different isokinetic joint velocities, unlike models which did not measure fascicle velocities e.g.^33^. *G* was constrained between 3 and 9, similarly to Brennan et al.^32^. Four participants were excluded from the force–length–velocity potential data because the model could not be fitted, and this variable was obtained from *n* = 17 participants. We used *L*_0_ to represent fascicle length in the correlation analyses.

### Data analyses and statistics

Changes in force, fascicle length, pennation angle and muscle length were calculated for 50 ms time intervals up to 150 ms after torque onset, i.e. 0–50 ms, 50–100 ms, and 100–150 ms. Fascicle velocity was calculated from fascicle length data using the central difference method and averaged over the time intervals (Fig. 1). RFD was defined as the change in force over each time interval. Architectural gear ratio was calculated by dividing muscle velocity by fascicle velocity^34^. To calculate when force during RFD tests reached the maximal force potential according to the force–length–velocity limits, the first timepoint at which participants reached 95% of the force–length–velocity potential (Eqs. 1–3) was used^20^.

**Figure 2:**
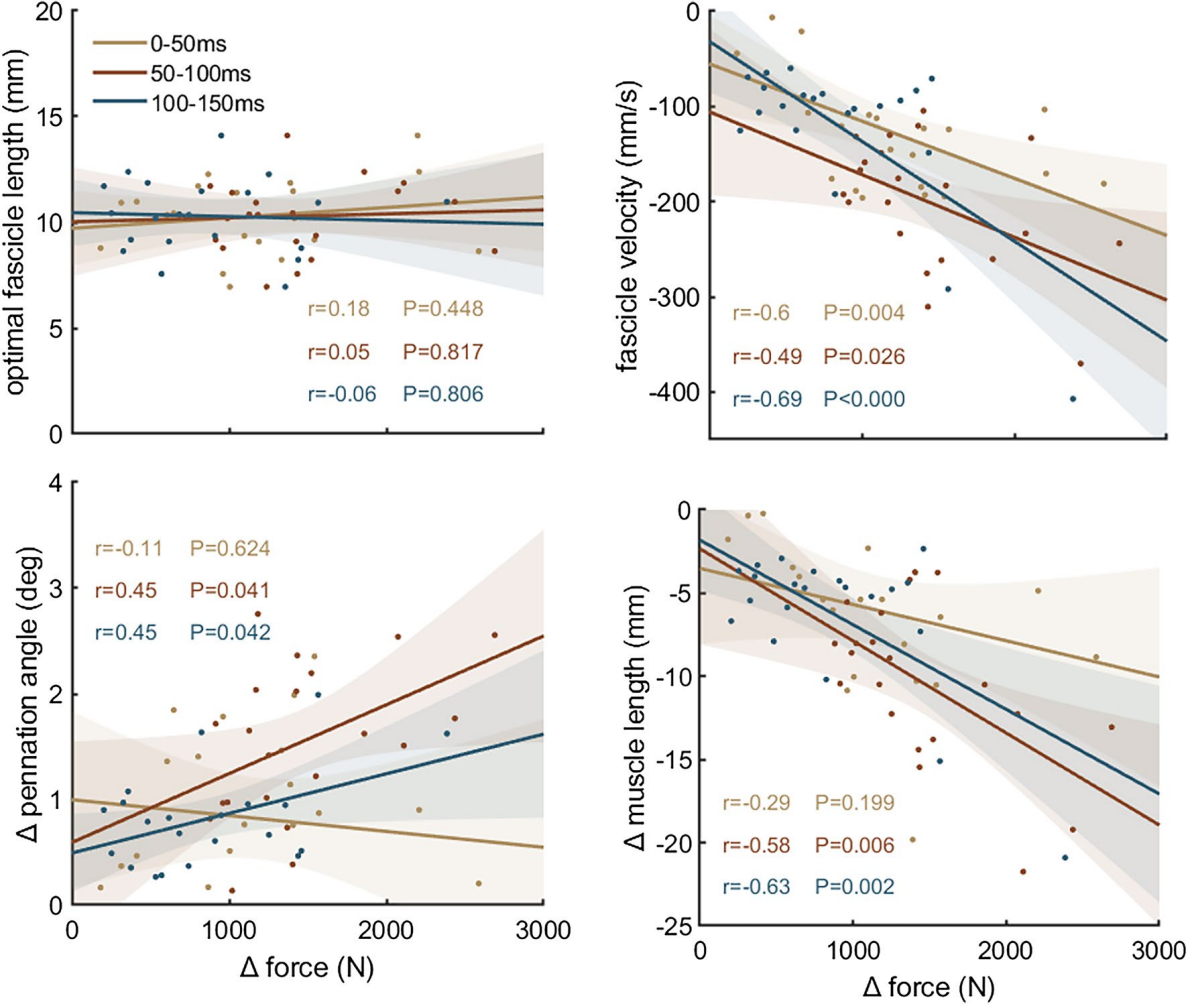
Correlation analysis between absolute force increase during rapid contractions and optimal fascicle length, fascicle velocity, change in pennation angle and muscle length change (n = 21). Optimal fascicle length was defined as fascicle length at the greatest force. Fascicle velocity, pennation angle and muscle length values were determined during the respective time intervals.

**Figure 3:**
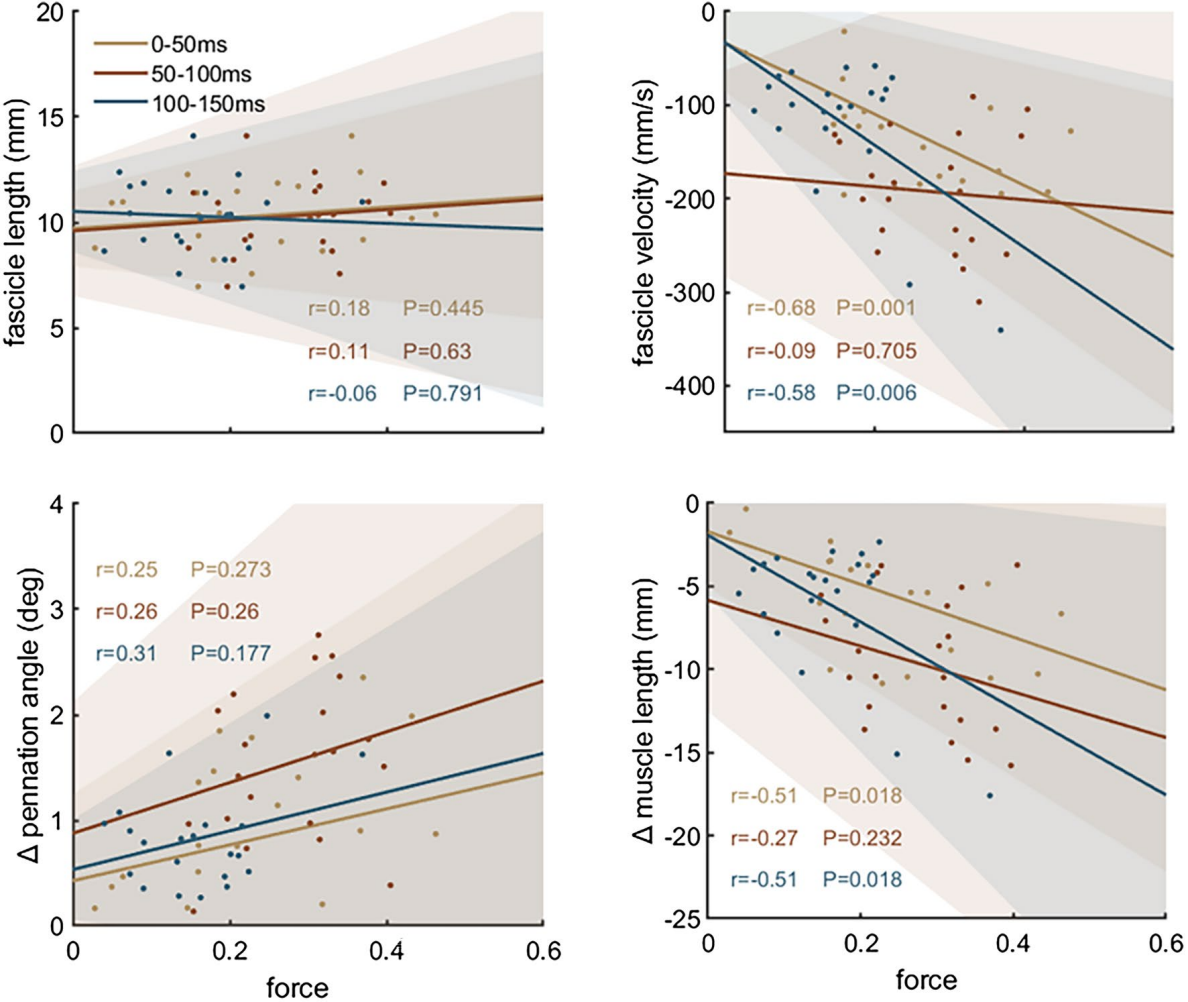
Correlation analysis between normalised force increase (to maximum force) during rapid contractions and optimal fascicle length, fascicle velocity, change in pennation angle and muscle length change (n = 21). Optimal fascicle length was defined as fascicle length at the greatest force. Fascicle velocity, pennation angle and muscle length values were determined during the respective time intervals.

**Figure 4:**
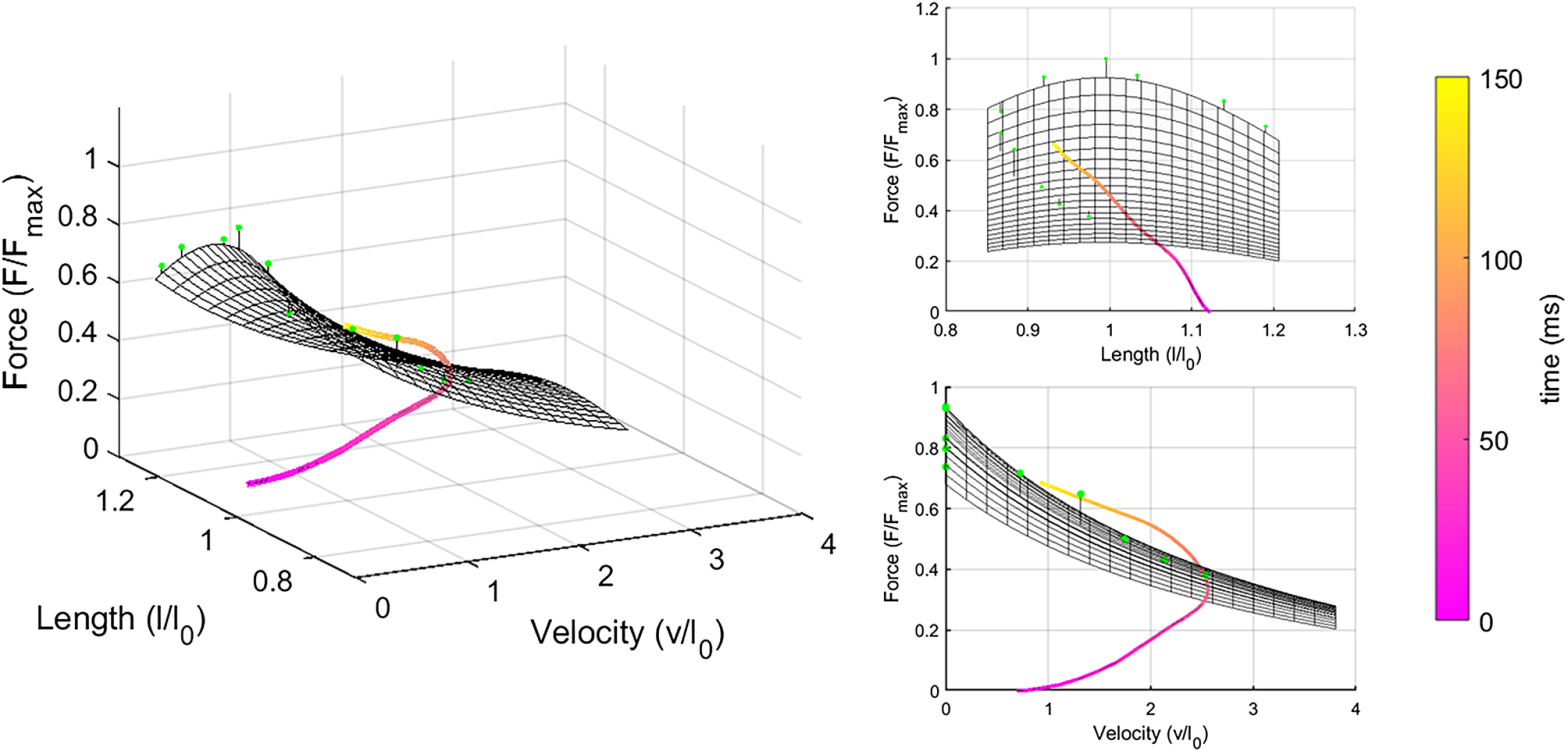
Normalised force–length–velocity potential (mesh), measured values during isometric and isokinetic contractions (green dots), and the relative force–length–velocity values during rapid contractions from force onset to 150 ms post onset for the vastus lateralis muscle shown as 3d-plot (left) and 2d-projections (right). Forces were normalised to maximum force; Fascicle lengths were normalised to optimal fascicle length. Data are mean values (n = 17).

Kolmogorov–Smirnov test was used to test the normality of the data distribution. The Pearson correlation coefficient was calculated to test the relation between optimal fascicle length and change in force during each time intervals. Pearson correlation coefficients were also calculated between dynamic architecture parameters, i.e. mean fascicle velocity, pennation angle change, muscle length change and architectural gear ratio, and absolute and normalised force for all intervals. Correlation coefficients were considered strong for r ≥ 0.7, moderate for 0.7 > r ≥ 0.4, and weak for r < 0.4^35^. MATLAB (R2018b, https://se.mathworks.com/) was used to conduct the statistical tests (with significance level α = 0.05) and to generate Figs. 1, 2, 3, 4.

During the rapid contractions, we calculated the time from contraction onset to 95% of *F*_max,_ according to Eq. (3).

## Results

During the RFD trials, force increased to 1180 ± 625 N after 50 ms, 2638 ± 944 N after 100 ms, and 3515 ± 950 N after 150 ms. Torque increased to 64 ± 30 Nm after 50 ms, 134 ± 47 Nm after 100 ms, and 173 ± 47 Nm after 150 ms. While force/torque increased, fascicles and total muscle length shortened, and pennation angle increased exponentially during all time intervals (Table 1). Correlations between force and muscle architecture parameters are shown in Fig. 2 and correlations between normalised force and muscle architecture parameters are shown in Fig. 3 for all time intervals. Optimal fascicle length was not related to force increase to 150 ms (*r* = 0.11, *P* = 0.632) or to force increase at any time interval for absolute and normalised force. There were also no significant correlations between pennation angle and force increases (*r* = 0.13–0.34, *P* > 0.1). Fascicle shortening velocity was negatively correlated with force increase for all intervals and for the whole period (0–150 ms; r = 0.52, P = 0.016), i.e. fascicles shortened faster when force change was greater. Normalised force was also correlated with fascicle velocity but only for 0–50 ms and 100–150 ms after force onset. Absolute force and pennation angle change were positively correlated, i.e. they changed more in participants with greater force change, during 50–100 ms and 100–150 ms after force onset but pennation angle change did not correlate with force during the first 50 ms or for the whole period (0–150 ms; *r* = − 0.01, *P* = 0.701). Correlations of normalised force and pennation angle changes were also positive but not significant. Muscle shortening also positively correlated with absolute force increase for 50–100 ms and 100–150 ms intervals and with relative force increase for 0–50 and 100–150 ms. The correlation for the whole period was not significant (*r* = 0.40, *P* = 0.072). AGR was not correlated with force for any interval (0–50 ms: *r* = − 0.31, *P* = 0.208; 50–100 ms: *r* = − 0.07, *P* = 0.793; 100–150 ms: *r* = − 0.07, *P* = 0.769) or the whole period (0–150 ms; *r* = − 0.24, *P* = 0.340). AGR was also not correlated to normalised force (0–50 ms: *r* = − 0.07, *P* = 0.771; 50–100 ms: *r* = 0.11, *P* = 0.654; 100–150 ms: *r* = − 0.06, *P* = 0.819). During the rapid contractions, participants reached 95% of their individual force–length–velocity potential after 62 ± 24 ms (Fig. 4).

**Table 1:** Changes in force, fascicle length, pennation angle and muscle length in 50 ms time intervals after torque onset.

	0–50 ms	50–100 ms	100–150 ms
Δ Force (N)	+ 1180 ± 625	+ 1458 ± 505	+ 877 ± 548
Δ Fascicle length (mm)	− 5.3 ± 3.4	− 14.5 ± 5.9	− 20.5 ± 7.8
Δ Pennation angle (°)	+ 0.8 ± 0.8	+ 2.4 ± 1.2	+ 3.2 ± 1.2
Δ Muscle length (mm)	− 6.1 ± 4.7	− 16.5 ± 8.3	− 22.7 ± 10.0
Architectural gear ratio	+ 1.06 ± 0.07	+ 1.05 ± 0.06	+ 1.03 ± 0.02

## Discussion

In this study, we aimed to investigate the relationships between muscle architecture, contractile behaviour and rate of force development during rapid voluntary contractions. Against our hypothesis, RFD was not correlated to optimal fascicle length during the first 150 ms of contraction, nor to any of the 50 ms sub-intervals within this range. On the other hand, variables reflecting contractile dynamics were related to absolute and normalised RFD in certain time intervals. Fascicle shortening velocity was related to all discrete time intervals of RFD that we analysed, until 150 ms. Changes in whole muscle length and in pennation angle correlated with RFD between 50 and 100 ms and RFD between 100 and 150 ms, although AGR was not related to RFD for any time interval. At the individual level, our estimations of force–length–velocity potential support the influence of fascicular behaviour on RFD, as maximal force potential was reached 62 (± 24) ms after force onset.

The present lack of correlation between RFD and fascicle length is consistent with the findings from a recent study^19^, despite broad differences between methodological approaches. In their study, Maden-Wilkinson and colleagues ascribed the lack of correlation to the low individual variability of their cohort, which had an unusually low standard deviation (10 mm) for fascicle length measurements compared to previous studies (e.g. Blazevich et al.^36^; see discussion on page 1107). This explanation seems less likely for our fascicle length data, for which the standard deviation was 18 mm. We speculate that the lack of correlations between fascicle length and RFD may be attributed to several physiological and methodological factors. Inter-individual variability in physiological factors affecting fast force production such as faster fibre conduction velocity^37^ muscle fibre type^38^ may affect the relative importance of the fibre length. Another reason could be related to how accurate fascicle length reflects the number of in-series sarcomeres, which may vary along fibres and the muscles^39^. Amongst methodological shortcomings are the necessary extrapolation of fascicle trajectories, the omission of the three-dimensional fascicle curvature during two-dimensional scans and in the analysis, or variations of architecture within the muscle, which simplify the three-dimensional structure of the muscle and reduce the validity of architecture measurements^40,41^. Similar factors likely influenced the correlations between pennation angle change and RFD, which were all non-significant in our study. The lack of association with pennation angle also suggests that the reduction of force related to the fascicle angulation in pennate muscles plays a minor role for explosive RFD, as suggested in a recent retrospective discussion on this topic^15^. Force reductions due to fascicle orientation are relatively small (− 3.4% for an angle of 15°) and may not affect RFD substantially with the sensitivity of the current methods. Overall, the lack of relationship between static muscle architecture measurements and RFD indirectly supports the dominant influence of neural factors on rapid force production^8,42^ and highlights the limitations of current in vivo architecture measurements when used as a proxy to sarcomeres number. These findings are in line with a recent review investigating the effect of actively induced changes in fascicle length on muscle mechanical function. Conclusive evidence for a relationship between fascicle length and shortening velocity seems lacking from the current literature^43^.

In contrast to fascicle length, our data indicate an association between fascicle dynamics and RFD within each 50 ms time intervals. Greater RFD was related to higher fascicle shortening velocities, with moderate correlations for the three assessed time intervals. These results from fixed-end contractions fit with recent findings of a similar relationship during isometric contractions with submaximal loading rate, concomitant with an increased neural drive^17^. Contrary to isokinetic contractions used in some of the before mentioned studies^44^, our fixed-end contraction measurements start from a resting position with no prior activation. The activation is maximal from around 50 ms^45^, while fascicles shortening velocity increases over a longer period, causing a loss of fascicle force production capacity. In addition, fascicle rotate about their insertion, decreasing the contribution of fascicle shortening to muscle shortening (see “Discussion” about AGR). However, these data confirm that fascicle contractile velocity is linked to RFD, also before reaching maximal force during maximal contractions. The strength of the relationships between RFD and fascicle velocity is affected by inter-individual variability in intrinsic factors, such as myosin kinetics^46^ and fiber type composition^38^, or stiffness of in-series and in-parallel connective tissue^47^. We speculate that the relative contribution of factors determining RFD is not constant and may explain for example why correlations between fascicle velocity and RFD were lowest (or lacking when force was normalised) for the 50–100 ms interval. A combined analysis of all these factors is required for a finer analysis. Despite the dependency of force on multiple factors, correlation analyses revealed that about 48% and 34% of the variation in late RFD could be explained by fascicle shortening velocity for absolute and normalized force, respectively.

In addition to fascicle shortening, changes in pennation angle contribute to the shortening of pennate muscles through rotation of fibres about their insertions^48^. We therefore expected that architectural gearing would be positively related to RFD. Despite the moderate correlations seen with fascicle rotation and whole muscle shortening in later time-intervals, AGR was not correlated to RFD in the present study, which contrasts with a recent study reporting a positive relationship, between gastrocnemius medialis AGR and explosive torque, using ultrafast ultrasound^13^. Albeit speculative, the discrepant results could be explained by a higher series elastic compliance of the plantar flexors compared to the knee extensors, which allows greater muscle shortening in fixed-end contractions of the plantar flexors. Alternatively, ultrasound sampling frequency may have been too low in our study to calculate AGR precisely enough during fast contractions, with ~ 4–6 images per 50 ms interval. Although we did not find the expected correlations between RFD and AGR, the correlation with fascicle rotation and muscle shortening, and the fact that AGR was highest in the beginning of the contraction are noteworthy.

Hager et al. had shown that force–velocity properties are a limiting factor for RFD from ~ 100 ms after the onset of force in the gastrocnemius medialis^20^. For the vastus lateralis we found that force–length–velocity properties were also limiting earlier during the contraction (after 62 ± 24 ms; 95% CI) compared to the gastrocnemius. Broad differences in methodological approaches do not allow direct comparisons but suggest that the force–velocity relationship may constrain force increase earlier in the knee extensor muscles than in plantar flexors. This would be coherent with the smaller deformation of the patellar tendon compared to that of the gastrocnemius sub-tendon^49^. Alternatively, the influence of methodological differences between studies such as the different timing of force–velocity constraints cannot be excluded. Our RFD tests focused on fast contractions, without reaching maximal torque. Since RFD differs between this testing modality and fast contractions aiming at maximal torque^23^, time-course and amplitude of activation may be dissimilar in the study by Hager and colleagues and the present one. A difference in the calculation method may also partly explain differences between the two studies. When we base our calculation on group mean values, similar to Hager and colleagues, the delay for the force to reach force–length–velocity potential during the RFD contraction is offset to 80 ms. Additionally, we measured fascicle velocity at five isokinetic joint velocities instead of maximum shortening velocity and added information about operating length due to its influence on force production.

Our approach to model muscle properties has several shortcomings that may explain why our RFD data slightly exceeds the force–length–velocity potential (Fig. 4). Firstly, force–length properties may be affected by a mismatching of operating lengths between isometric and dynamic contractions. Matching fascicle lengths in different tests is methodologically difficult, because of differences in elastic tissue deformation at different force levels. For contractions with higher forces (i.e., isometric and slow isokinetic contractions) elastic tissue strain is higher, resulting in shorter fascicle operating lengths. The operating length difference between the fastest and slowest isokinetic condition is for example 12%. In addition, RFD tests start at longer fascicle length, relative to isokinetic tests, because of a greater slack. Our assumption of a constant force contribution of the vastus lateralis muscle to the knee torque is based on supportive evidence^50^. However, we cannot rule out that inter-individual variability in several factors would affect the relation between the force of this muscle and the joint angle, adding noise to our results based on force estimation. Because of these methodological considerations, the temporality of the threshold set by force–length–velocity properties should be considered specifically to the present testing conditions and interpreted with caution. The lower value of the estimated force potential compared to the force recorded during RFD could indicate an underestimation of the force capacity. If force capacity is underestimated, the limiting force–length–velocity properties could therefore be reached later than our model shows.

In conclusion, our data shows that knee extensor RFD is positively correlated to vastus lateralis fascicle shortening velocity and, after the first 50 ms of contraction, to the amount of fascicle rotation and whole muscle shortening—but not to AGR. These in vivo observations confirm the theoretical importance of muscle contractile behaviour for fast force production, even during an “isometric” contraction at the joint level. Moreover, the analysis of the individual vastus lateralis force–length–velocity properties indicates that these are limiting for RFD from on average 62 ms after contraction onset, supporting the hypothesis that these properties are limiting for mid- to late-RFD^20^. The lack of correlations between RFD and static measures of muscle architecture indicates that our measurements did not reflect sarcomere arrangement accurately enough, although they reached a sufficient level of validity for dynamic contractile behaviour. The complexity of the neuro-musculo-tendinous system and the current methodological limits may prevent to observe such a correlation.

## Data Availability

The datasets generated during and/or analysed during the current study are available from the corresponding author on reasonable request.
